# Real-Life Performance of Mepolizumab in T2-High Severe Refractory Asthma with the Overlapping Eosinophilic-Allergic Phenotype

**DOI:** 10.3390/biomedicines10102635

**Published:** 2022-10-19

**Authors:** Ruperto González-Pérez, Paloma Poza-Guedes, Elena Mederos-Luis, Inmaculada Sánchez-Machín

**Affiliations:** 1 Allergy Department, Hospital Universitario de Canarias, 38320 Tenerife, Spain; 2 Severe Asthma Unit, Hospital Universitario de Canarias, 38320 Tenerife, Spain

**Keywords:** severe asthma, phenotype, T2-inflammation, mepolizumab, allergen exposure, mites

## Abstract

Severe asthma (SA) is categorized into multiple overlapping phenotypes and clinical characteristics driven by complex mechanistic inflammatory pathways. Mepolizumab is a human monoclonal antibody effectively targeting interleukin-5 in severe eosinophilic asthma. However, the eligibility of biologics in coincident SA phenotypes is still unclear. We assessed the efficacy and safety of mepolizumab in real-life patients with the overlapping T2-high SA endotype. This was a phase IV, single-centre observational cohort study including patients with severe refractory T2-high asthma in treatment with mepolizumab. After 12 months of treatment with mepolizumab, significant improvements (*p* < 0.0001) in asthma control and lung function were recorded. Rates of clinically significant annual asthma exacerbation were also decreased by 71.22% after 52-week therapy with mepolizumab (*p* < 0.001) associated with a reduction in the mean daily dose of oral corticosteroids. Two patients (3.27%) had to discontinue mepolizumab due to musculoskeletal disorders with no severe safety issues reported. The use of mepolizumab as an add-on therapy in routine clinical practice was safely associated with significant clinical and functional in the overlapping eosinophilic-and-allergic SA phenotype. The current data should support clinical and therapeutic decision-making in this T2-high SA endotype.

## 1. Introduction

Severe asthma (SA) is a complex heterogeneous condition characterized by chronic inflammation of the pulmonary airways that affects 3–10% of the asthma population worldwide, comprising several pheno-endotypes that are distinguished by different clinical characteristics, pathobiological mechanisms, and biomarker expressions [1,2]. Considering the pathogenesis of SA, lung inflammation principally falls into a dichotomy of a preponderant type 2-high response—including eosinophilic, allergic, and non-allergic asthma—and a type 2-low response involving a neutrophilic and pauci-granulocytic SA. [3,4]. Since a broader proportion of cases of SA are predominantly type 2–high asthma (>50%), the concurrence of both eosinophilic and neutrophilic airway inflammation has also been documented as mixed granulocytic asthma [5]. In addition, despite the fact that an overlap of prevalent subtypes of type 2 inflammation has also been described in an unselected population, the frequency and degree of coexistence among these subtypes remains unclear [6,7].

House dust mites (HDM) and storage mites (SM) allergens are nowadays considered key players in the pathogenesis and severity of type 2-high inflammatory responses in asthma [8,9]. HDM allergens induce Th2 immunity through the respiratory epithelium, resulting in the production of alarmins -IL-25, IL-33, and thymic stromal lymphopoietin (TSLP)- that stimulate release of IL-4, IL-5, and IL-13, inducing the production of sIgE, the eosinophil recruitment and survival in tissues, the production of mucus, the modulation of the airway smooth muscle contraction and airway hyperresponsiveness, finally leading to lung tissue remodeling [10,11,12].

As monoclonal antibodies (mABs) have become available, the inflammatory endotype characterization of SA turned out to be crucial to provide a more personalized workup and management when considering biologic therapy to treat patients with uncontrolled SA more efficiently [13]. According to recent guidelines, some SA patients who display overlapping phenotypic characteristics are currently eligible for five different biologicals -anti-IgE therapy, three anti–IL-5 biologics, and one anti–IL-4R biologic- depending on the eligibility criteria used [14,15]. Regrettably, despite that fact that many patients with SA concurrently qualify for several biologics, no head-to-head trials have been conducted at present to aid clinicians in this task [16].

Mepolizumab is a subcutaneously (SC), monthly-administered humanized IL-5 antagonist mAB, approved in Spain in 2016 for the treatment of patients with severe refractory asthma inadequately controlled with standard high-dose inhaled corticosteroids (ICS) and other long-term controller agents [17,18]. Although the efficacy and safety of mepolizumab has been widely demonstrated in severe eosinophilic asthma in previous randomized controlled trials (RCT) and real-world studies (RWE), the role of mepolizumab has not been fully assessed in populations with co-existing severe eosinophilic and allergic asthma [19,20]. In the Canary Islands, a geographical area leading in the national prevalence of asthma and number of prescribed biologics for SA in Spain [21,22], the coexistence of overlapping type-2 inflammation subtypes—eosinophilic and allergic—in moderate and SA have been formerly described [23,24]. Hence, this study was conducted to investigate the real-world performance of monthly SC mepolizumab 100 mg after 52-weeks of treatment in patients with the coexisting allergic-eosinophilic refractory SA phenotype.

## 2. Materials and Methods

### 2.1. Subjects

The present phase IV, single-centre observational cohort study included 61 subjects with severe uncontrolled asthma in treatment with mepolizumab 100 mg every 4 weeks (100 mg-q4w) for 52 consecutive weeks in the Severe Asthma Unit and Outpatient Allergy Clinic at Hospital Universitario de Canarias (Tenerife, Spain). Key inclusion criteria to start treatment with mepolizumab were a clinician-confirmed diagnosis of severe refractory asthma with the T2 signature—i.e., eosinophilic; high total IgE and sIgE to airborne allergens—according to the 2020 Global Initiative for Asthma (GINA) Guidelines [25] in subjects aged >12 years with ≥2 asthma exacerbations (AE) during the previous year with use of oral corticosteroids (OCS), despite receiving appropriate treatment for the degree of their asthma severity or steroid dependence and a regular clinical follow-up since the biologic therapy was started (every 3-to-6 months).

The following data were retrospectively collected from patients’ clinical records from January 2021 to July 2022, over an 18-month period: Sociodemographic data, clinical profile—age of asthma onset, atopy, nasal polyps, or any relevant comorbidity to asthma—, number of asthma exacerbations, and concomitant use of systemic corticosteroid therapy. The validated self-administered Asthma Control Test (ACT) and Sino-Nasal Outcome Test-22 (SNOT-22) questionnaires were assessed and pulmonary function, including pre- and post-bronchodilator spirometric tests—Datospir 600^®^, Sibel S.A.U., Barcelona, Spain- and fractional exhaled nitric oxide (FENO), Fenom Pro^®^, Caire Diagnostics, Ball Ground, GA, USA—were performed according to standardized guidelines [26,27].

Clinically significant AE were considered as those demanding systemic steroids for at least 3 days or doubling the dose in those subjects on maintenance OCS, or if the patient had visited an emergency department or was admitted to hospital. Corticosteroid dependence was defined as the daily use of OCS for at least 6 months. Asthma exacerbation rates, ACT, SNOT-22, use of systemic steroids, pulmonary respiratory function, and FENO were compared from baseline to 52-weeks values after subsequent treatment with mepolizumab 100 mg-q4w. Pregnant and breast-feeding women were excluded. The study was approved by the local Ethical Committee of our Institution and informed consent was signed by all subjects and parents/guardians for those participants <18 y.o.

### 2.2. Skin Prick Test

The skin prick test (SPT) was performed according to European Guidelines, enclosing a diagnostic panel (Diater, Madrid, Spain) with standardized *Dermatophagoides pteronyssinus* (*D. pteronyssinus*), *Dermatophagoides farinae* (*D. farinae*), and *Blomia tropicalis* (*B. tropicalis*) mite extracts [28]. Histamine (10 mg/mL) and saline were used as positive and negative controls as usual. Following everyday practice, antihistamines were withdrawn a week prior to the SPT, and wheal diameters were immediately measured after 20 min; those greater than 3 mm were regarded as positive.

### 2.3. Blood and Serological Analysis

Serum blood samples including blood eosinophil measurement were obtained from all participating subjects, identified with a code label, stored at −40 °C, and thawed immediately before the in vitro analysis. Total IgE levels, specific IgE (sIgE) to *D. pteronyssinus*, *D. farinae*, and *B. tropicalis* (raw extracts), and sIgE to Der p 1, Der p 2, Der p 5, Der p 7, Der p 10, Der p 11, Der p 20, Der p 21, Der p 23, Blo t 5, Blo t 10 and Blo t 21 (ALEX MacroArray Diagnostics, Vienna, Austria) according to the manufacturer’s instructions. In brief, ALEX is a multiplex array containing 282 reagents (157 extractive allergens and 125 molecular components). The different allergens and components are coupled onto polystyrene nano-beads, and then the allergen beads are deposited on a nitrocellulose membrane, as published elsewhere [29].

Total IgE levels were expressed in international units per unit volume (IU/mL), sIgE levels were expressed in kU_A_/L. Values ≥ 0.35 kU_A_/L were considered positive. Blood eosinophils, total IgE levels, specific sIgE to *D. pteronyssinus*, *D. farinae*, and *B. tropicalis* were compared after 52 weeks of treatment with mepolizumab 100 mg-q4w to baseline values.

### 2.4. Statistical Analysis

Demographic features were summarized by medians and standard deviations for continuous variables and percentages for categorical variables. A paired two-tailed t-test was used for comparison of before/after periods for continuous variables and the McNemar test for categorical variables. To compare more than two groups, ANOVA was used. A P value of less than 0.05 was considered statistically significant. All statistical data were analysed using GraphPad Prism version 8.0.0 for Windows, GraphPad Software, La Jolla, CA, USA.

## 3. Results

### 3.1. Demographic and Clinical Characteristics of Subjects at Baseline

**Figure 1:**
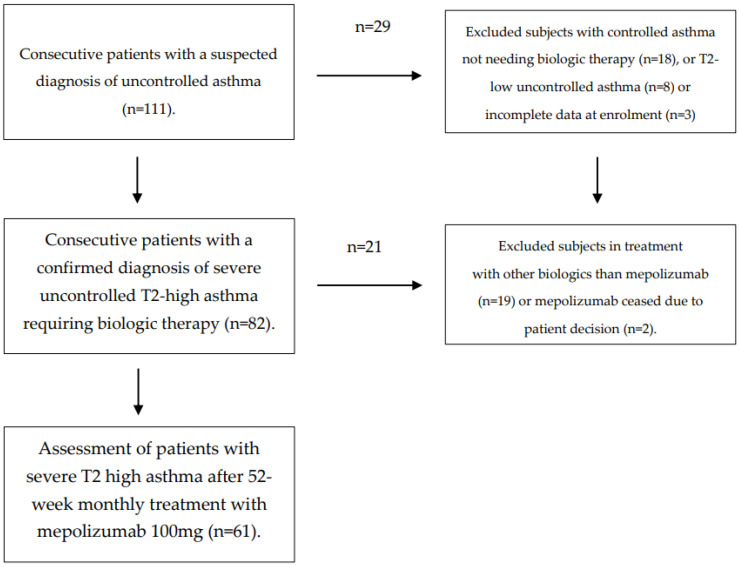
Flow diagram of patients and study selection.

A total of 111 patients were screened, with 61 of them—37 females, 24 males, median age 49.0 years—finally confirming their eligibility for the study (Figure 1).

Up to 83.6% of the individuals fulfilling the GINA criteria for non-occupational severe uncontrolled T2 asthma showed a positive SPT to *Dermatophagoides* spp. and/or *B. tropicalis* and had their asthma onset during childhood or adolescence −42 out of 61 patients, 68.85%—with a high (80.32%) known family history of atopy reported. Regarding main comorbidities, 52 patients (85.24%) suffered from allergic rhinitis, 22 subjects (36.06%) had nasal polyposis, NSAID sensitivity was present in 11 individuals (18.03%), 17 of them (27.86%) suffered from chronic rhinosinusitis (CRS), gastroesophageal reflux disease (GERD) affected 15 out of 61 subjects (24.59%), and atopic dermatitis was confirmed in 14.75% of the studied population. The mean body mass index (BMI) was 29.10 ± 4.04 kg/m, with 25 (40.98%) and 24 (39.34%) patients being obese or overweight, respectively (Table 1).

All patients showed poor asthma control (ACT score < 20) at baseline with a mean value of 13.08 ± 4.61. The mean number of annual clinically relevant asthma exacerbations was 3.37 ± 1.34, and daily OCS were needed in 10 (16.39%) patients with a mean maintenance dose of 11.3 ± 9.6 mg/day. Regarding previous biologic treatments, a total of 11 (18.3%) out of 61 subjects were switched from omalizumab to mepolizumab due to inadequate symptom control or limited clinical response.

### 3.2. Baseline Blood Eosinophils and Total IgE

Blood eosinophils showed a mean value of 541 ± 283.04 eosinophils/μL at baseline. A total of 19 out of 61 patients (31.14%) showed an overall mean baseline blood eosinophil count of 876.1 ± 242.98 cells/µL. In addition, a quantitative analysis of serum total IgE was performed to evaluate the atopic status in the study population with a mean value of 614.53 ± 1196 IU/mL at baseline.

### 3.3. Prevalence, sIgE Reactivity and Individual Molecular Profile in Serum sIgE from Severe Uncontrolled Asthmatic Subjects

Fifty patients (81.96%) were independently sIgE positive (≥0.35 kU_A_/L) for either the raw extract of *D. pteronyssinus, D. farinae* or *B. tropicalis*, showing a corresponding mean value of 48 ± 78.68, 50 ± 81.96 and 28 ± 45.9 at baseline.

Considering the prevalence of individual mite molecular allergens in this subset, sensitization to Der p 2 was most frequently identified in 41 out of 50 patients (82.0%), followed by Der p 1 (75.0%), Der p 23 (72.22%), Der p 5, Der p 21 (58.33%), Der p 7 (47.22%), Blo t 5 (41.23%), and Blo t 21 (35.33%). Minor allergens—i.e., Der p 10, Blo t 10, Der p 11, and Der p 20—were represented in less than 12% of the present sample. The quantification (mean ± SD) of single molecular allergens was led by Der p2 (17.69 ± 16.51), Dep p 23 (12.62 ± 14.59), and Der p1 (10.47 ± 5.98).

### 3.4. Assessment of Clinical Severity, Asthma Exacerbations, Pulmonary Function, and T2 Inflammation Biomarkers after Therapy with Mepolizumab 100 mg-q4w for 52 Weeks

**Figure 2:**
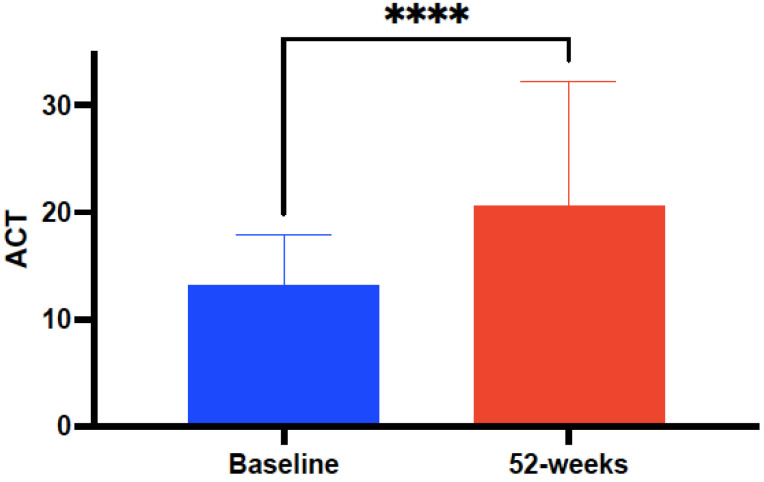
Evolution of asthma control measured by the Asthma Control Test (ACT) in subjects with severe refractory asthma with the eosinophilic–allergic phenotype, before and after 52-week treatment with mepolizumab 100 mg every 4 weeks. Asterisks indicate statistical significance (**** *p* < 0.001).

**Figure 3:**
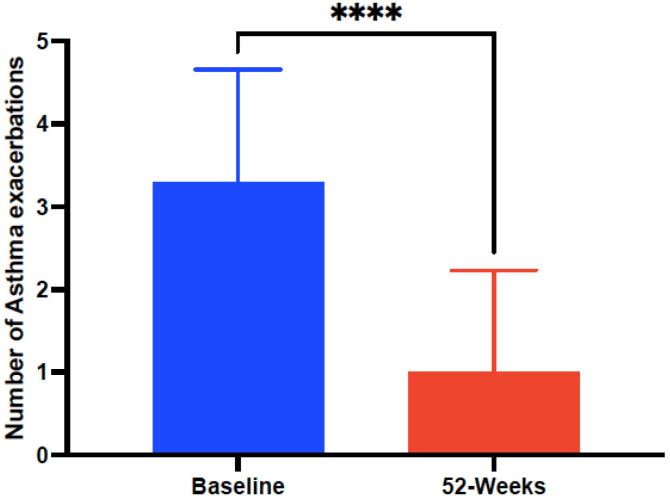
Evolution of number of annual clinically relevant asthma exacerbations before and after 52-week treatment with mepolizumab 100 mg every 4 weeks. Asterisks indicate statistical significance (**** *p* < 0.001).

Clinical, functional, and laboratory data were statistically compared at baseline and after 52 weeks of treatment with mepolizumab 100 mg-q4w, as shown in Table 2. The mean ACT score at baseline significantly (*p* < 0.0001) improved from 13.8 ± 4.61 to 18.91 ± 4.79 after 52-weeks of treatment with mepolizumab 100 mg-q4w (Figure 2). Significant improvements (*p* < 0.001) were also observed in the mean number of annual AE rates from 3.37 ± 1.34 at baseline to 0.97 ± 1.19 after mepolizumab (Figure 3) and also in the number of individuals requiring daily use of OCS—from 10 (16.39%) patients at baseline to 2 (3.27%) after 52 weeks of treatment with mepolizumab—and a reduction in the mean OCS maintenance dose of 4.8 ± 5.4 mg/day (from 11.3 ± 9.6 to 6.5 ± 4.2 mg/day).

**Figure 4:**
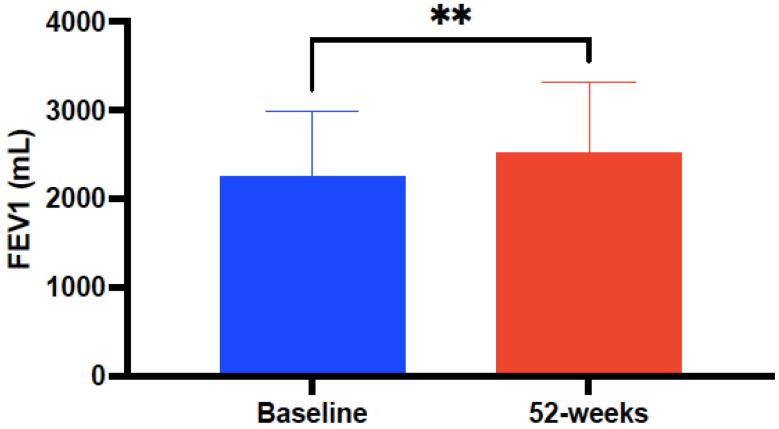
Evolution of pulmonary function (FEV1 in mL) before and after 52-week treatment with mepolizumab 100 mg every 4 weeks. Asterisks indicate statistical significance (** *p* < 0.01).

Pulmonary function—FEV1, measured both in mL and as a percentage—was significantly increased up to 318 ± 150 mL (*p* < 0.0001), from 72.75 ± 17.85 at baseline to 80.95 ± 18.17 (*p* = 0.0134) after treatment with mepolizumab (Figure 4). In contrast, no significant changes were observed in FENO (ppb) after 52 weeks of mepolizumab therapy compared to baseline values (57.51 ± 47.59 versus 53.35 ± 33.46, *p* = 0.341).

**Figure 5:**
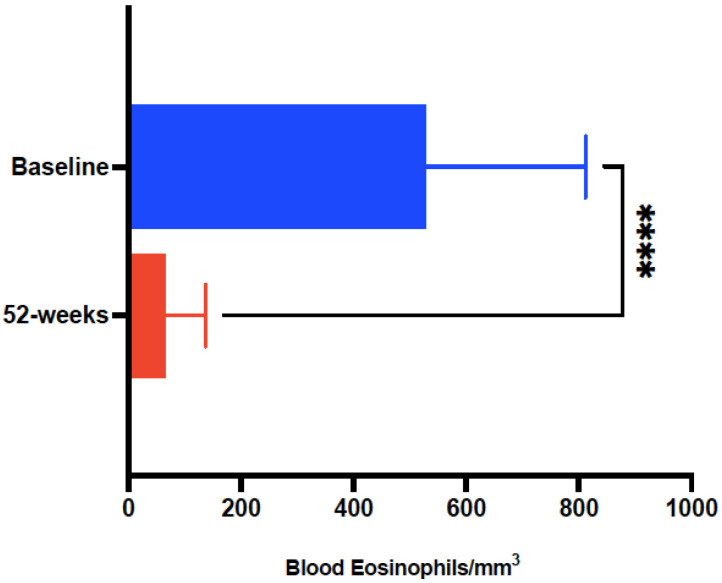
Evolution of absolute blood eosinophils in subjects with severe refractory asthma with the eosinophilic-allergic phenotype, pre-treatment and after 52-week treatment with mepolizumab 100 mg every 4 weeks. Asterisks indicate statistical significance (**** *p* < 0.001).

Laboratory findings confirmed a marked reduction in the absolute blood eosinophil counts (cells/µL) after 52-week treatment with mepolizumab (541 ± 283 vs. 59.19 ± 48.95, *p* < 0.0001) (Figure 5), with no significant changes for total IgE or sIgE to *D. pteronyssinus, D. farinae* or *B. tropicalis*.

In addition, improvements in asthma control, pulmonary function, and rate of annual AE were confirmed after 52 weeks of mepolizumab, irrespective of the blood eosinophil threshold (<600 cells/µL vs. ≥600 cells/µL) at baseline (Table 3).

Improvements in asthma control (*p* = 0.008) and number of clinically relevant asthma exacerbations (*p* = 0.002), but not in FEV1 (*p* = 0.104), were observed in the subgroup of subjects (*n* = 11) switched from omalizumab to mepolizumab despite the fact that a significant reduction in blood eosinophils (*p* = 0.002) was confirmed after the 52-week treatment with mepolizumab compared to baseline values.

### 3.5. Subgroup Analysis of Patients with Nasal Polyposis

A total of 22 (36.06%) out of 61 subjects—54% males, median age 48.5 years—had a concomitant diagnosis of nasal polyposis (NP), with 9 (14.75%) of them associating NSAID-Exacerbated Respiratory Disease (NERD). No significant differences were found in this subset compared to patients without nasal polyps in terms of mean levels of basal blood eosinophils (591.18 ± 322.44 versus 501 ± 239, *p* = 0.2215), serum total IgE (295.44 ± 344.05 versus 796.3 ± 1441.36, *p* = 0.0719), or sIgE to *D. pteronyssinus, D. farinae* or *B. tropicalis*.

**Figure 6:**
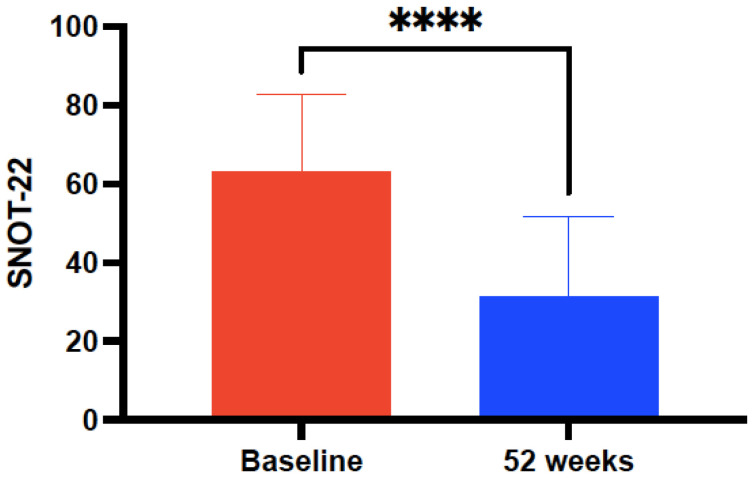
Evolution of Sino-Nasal Outcome Test-22 (SNOT-22) score in a subgroup of 22 patients with co-existing severe refractory asthma and nasal polyposis, pre-treatment and after 52-week treatment with mepolizumab 100 mg every 4 weeks. Asterisks indicate statistical significance (**** *p* < 0.001).

A greater improvement in terms of mean ACT score (7.3 ± 3 versus 5.39 ± 2.44, *p* = 0.0055), FEV1 (357 ± 37.95 versus 325 ± 2.44, *p* = 0.0067), but similar in the rate of AE (2.51 ± 0.43 versus 2.36 ± 0.09, *p* = 0.1492) was observed in the subset of patients with NP in relation to those without after 52 weeks of mepolizumab. The mean baseline SNOT-22 value showed a marked improvement after 52 weeks of mepolizumab in the subgroup of NP (63.75 ± 20.31 versus 33.5 ± 20.92, *p* < 0.0001) (Figure 6) in contrast to those patients without NP (45.95 ± 21.86 versus 42.76 ± 23.85, *p* = 0.0233). In addition, a significant decrease in the mean number of endoscopic sino-nasal surgeries for NP prior and after treatment with 52 weeks of mepolizumab was found (1.4 ± 0.9 versus 0.22 ± 0.42, *p* < 0.0001) (Table 4).

### 3.6. Sub-Analysis of Responders after 52 Weeks Treatment with Mepolizumab

A full response after the 52-week treatment with mepolizumab was considered when no AEs were recorded, ACT score ≥ 20, FEV1 ≥ 80% and OCS could be withdrawn [30]. According to these criteria, 17 out of 61 patients (27.86%) were regarded as super-responders (SR) and 44 subjects as partial responders (59.01%) or no-responders (13.11%) after 52 weeks of treatment with mepolizumab. Ten out of 17 (58.82%) super-responders—52.94% females, median age 44 years—had a concomitant diagnosis of NP with a similar basal ACT (14.88 ± 3.51 versus 12.38 ± 4.82, *p* = 0.0574) but a higher baseline FEV1 (2760 ± 529.19 versus 2099.09 ± 705.5, *p* = 0.0009) and fewer AE (2.7 ± 0.77 versus 3.63 ± 1.43, *p* = 0.014) at baseline compared to partial or no-responders. Regarding biological markers of T2-inflammation, no significant differences were found in the mean levels of basal blood eosinophils (630.35 ± 323.29 versus 507.27 ± 261.87, *p* = 0.1289), serum total IgE (1197.61 ± 2224.27 versus 415.06 ± 410.16, *p* = 0.423), sIgE to *D. pteronyssinus* (45.77 ± 51.57 versus 47.82 ± 50.55, *p* = 0.5125)*, D. farinae* (46.12 ± 50.34 versus 35.01 ± 45.53, *p* = 0.6953), or *B. tropicalis* (9.3 ± 29.4 versus 0.52 ± 2.08, *p* = 0.7046), in the subset of super-responders in respect to those regarded as partially or no-responder subjects.

### 3.7. Safety

Overall, 3 out of the 61 (4.91%) patients reported five adverse events related to mepolizumab during the 52-week study period. Two patients (3.27%) had to discontinue mepolizumab after 24 and 40 weeks of treatment with mepolizumab due to musculoskeletal and/or connective tissue—arthromyalgias—disorders. Both patients remained asymptomatic from their musculoskeletal disorders after discontinuation of mepolizumab and the subsequent 52-week follow-up. No severe adverse effects related to mepolizumab were recorded during the follow-up of the study (Table 5).

## 4. Discussion

As only a minority of patients with SA would be eligible for inclusion within a RCT, it has been proposed that those data could be complemented with RWE studies to achieve a broader understanding of the efficacy and safety of biological therapy under real-life conditions [31,32]. As the heterogeneity of T2 inflammation—thus supporting the co-existence of distinct subtypes of T2-high SA—has been previously addressed [33], we evaluated the real-life response after mepolizumab as add-on biologic therapy in 61 Caucasian patients with a coexisting allergic–eosinophilic uncontrolled SA phenotype.

As expected, recruited subjects associated a marked T2-inflammation co-morbidity spectrum, with a higher proportion of atopy (>83%) compared to MUSCA, mepolizumab, as adjunctive therapy in patients with severe asthma (46%), and REDES, Real world effectiveness and safety of mepolizumab (41.5%) studies, including allergic rhinitis (>85%), NP (36.06%) or atopic dermatitis (14.75%) [34,35]. Following ERS/ATS guidelines [36], baseline inflammatory biomarkers confirmed a preponderant Type-2 profile with 57 out of 61 patients (93.44%) showing a co-expression of at least 2 different biomarkers, i.e., blood eosinophilia (≥300 cells/µL), total IgE (≥150 UI/mL) and FeNO (≥25 ppb), of the T2 inflammatory trait. In addition, a positive sIgE to HDM and/or SM was observed in 33 out of 34 individuals, with concomitant increased levels of blood eosinophils, serum lgE, and FENO.

Notably, a widely heterogeneous molecular response to relevant mite allergens was identified in this cohort (>80%), including Der p1, a papain-like cysteine protease, which has a pivotal role in the maturation of HDM serine protease allergens that stimulates innate immune pathways, and Der p 2, belonging to the NPC intracellular cholesterol transporter 2, Niemann-Pick proteins type C2 family—which tends to be targeted by adaptive immune responses because of its auto-adjuvant characteristics [37,38,39].

Significant benefits were observed in the current population after the 52-week treatment with mepolizumab in several clinically relevant outcomes, including asthma control, lung function, annual rate of AE and use of OCS. In line with former post hoc meta-analysis of 2 RCTs, mepolizumab significantly improved ACT and FEV1, and reduced the rate of AE, regardless of blood eosinophil count at baseline [40,41], showing the efficacy of this therapy in subjects with the overlapping eosinophilic-and-allergic asthma phenotype. Intriguingly, despite that fact that mepolizumab did not reduce circulating IgE levels, our data are coincident with those speculating that IgE-mediated mechanisms are involved in releasing IL-5, as total serum IgE has been correlated with a reduction in AE in patients treated with mepolizumab [42].

Considering a minimally important difference (MCD) previously established at 3 points [43], the mean ACT score at baseline significantly improved 5.11±0.2 points after 52 weeks of mepolizumab 100 mg-q4w in the investigated population. Our results are in line with a recent systematic review by Charles et al., reaching a mean ACT improvement of 6.15 ± 1.01 points [44].

A higher reduction in the rates of clinically significant AEs (71.22%) was also confirmed in the selected cohort after 52 weeks with mepolizumab, compared to the pivotal MENSA—Mepolizumab treatment in patients with severe eosinophilic asthma—and MUSCA trials (up to 58%) and in subsequent RWE studies [34,45]. Furthermore, despite only a limited number (16.39%) of subjects were regarded as OCS-dependent, a significant reduction in the mean OCS maintenance daily dose of 4.8 ± 5.4 mg was confirmed, with 80% of them achieving of OCS discontinuation after 12 months of mepolizumab. The reduction in the use of daily OCS was also evaluated in 5 different RWE trials with mepolizumab, showing a comparable decrease in the mean daily dose of OCS (5.3 ± 2.2 mg) [44].

Considering pulmonary function, and 200 mL as the MCD for FEV1, our data confirmed a significantly higher improvement (>300 mL) compared to previous large clinical trials as MENSA (98 mL), SIRIUS—Steroid reduction with mepolizumab study—(128 mL), REDES (210 mL), and even in a post-hoc research considering therapy with mepolizumab according to omalizumab eligibility (114 mL) [35,44,45,46]. A possible explanation for such improvement in lung function in our population could be related to a better mean baseline FEV1% (72.75) value in respect to those published by Schleich and coworkers (67), MENSA (60), and analogous to REDES (70.12) [35,42,45]. In view of such findings, it could be hypothesized that the formerly demonstrated immunomodulatory effect of mepolizumab may significantly affect airway remodeling to a higher extent in those subjects with the overlapping allergic–eosinophilic SA phenotype [47,48].

Although the current SA cohort showed higher (541) mean blood eosinophils at baseline (cells/µL) compared to MENSA (290) [45] and Schleich et al. (476) [42], a lower reduction in mean blood eosinophils (−482) was found compared to a systematic review encompassing eight different trials (−609) after therapy with mepolizumab [44].

The clinical response to mepolizumab was also assessed in the present cohort, and despite different eligibility criteria being applied, a similar rate (>27%) of SR was obtained in comparison to a large Australian post-marketing surveillance registry of patients treated with mepolizumab for SA (31.34%) [49]. In line with this study by Harvey and coworkers, SR were predominantly younger females with a higher rate of NP, an improved pulmonary function at baseline, and fewer asthma exacerbations when compared to partial responders to mepolizumab.

Interestingly, despite it having been shown that NP increases the risk of mortality and impairs the quality of life in patients with SA and CRS [50], asthma control was improved to a greater extent in subjects with NP compared to those free from this condition. In agreement with the SYNAPSE study, a randomized, double-blind, placebo-controlled, phase 3 trial, most subjects eligible for repeat nasal surgery (19 out of 23 patients) showed a change of 9 points or more of improvement in the SNOT-22 score by week 52 and a significant decrease in their annual nasal surgery rates compared to baseline values. [51]

Besides the size of the studied population, the present real-life investigation has some limitations, as sputum induction was not assessed, and the lack of a control group must be considered when interpreting the overall data. Moreover, a longer follow-up period (>52-weeks) would be required to further establish the efficacy and safety of mepolizumab in this SA cohort.

## 5. Conclusions

Our results confirm that the use of mepolizumab as an add-on therapy in daily clinical practice for 52 weeks was associated with significant improvements in asthma control and lung function, and reductions in clinically relevant exacerbations in patients with the overlapping eosinophilic-and-allergic SA phenotype. In addition, treatment with mepolizumab was well tolerated, showing a safety profile coincident with former clinical investigations, providing clinically valuable insights to improve SA management in the studied population.

## Figures and Tables

**Table 1:** Demographic and clinical characteristics of patients prior to commencement of mepolizumab.

Variable	Severe Uncontrolled Asthma
*n* = 61 (%)	61 (100)
Age (y.o.) mean (SD)	45.98 (38.89)
<20 y.o. (%)	5 (8.19)
>20 y.o. (%)	56 (91.81)
Sex (Female%/Male%)	60.65/39.35
BMI mean (SD)	29.10 (4.04)
Smoking	
Never smoker	41 (67.21)
Former smoker	17 (27.86)
Smoker	3 (4.91)
Bronchiectasis (Chest CT Scanner)	3 (4.91)
SPT+ HDM and/or SM	51 (83.6)
Allergic Rhinitis (%)	52 (85.24)
Atopic Dermatitis (%)	9 (14.75)
Nasal Polyposis (%)	22 (36.06)
NERD (%)	11 (18.03)
Chronic Rinosinusitis (%)	17 (27.86)
Eosinophilic Esophagitis (%)	2 (3.2)
Food Allergy (%)	2 (3.2)
Asthma Onset at Childhood (%)	42 (68.85)
Family History of Atopy (%)	49 (80.32)

SD: Standard deviation of the mean. SPT: Skin Prick Test. HDM: House dust mites (*Dermatophagoides* spp.) SM: Storage mites (*Blomia tropicalis*). NERD: NSAID-exacerbated respiratory disease.

**Table 2:** Analysis of patients with refractory severe asthma with the coincident eosinophilic–allergic phenotype (*n* = 61) at baseline and after 52-week treatment with subcutaneous mepolizumab 100 mg every 4 weeks.

Variables	Baseline	After 52-Weeks of Mepolizumab	*p*
ACT *	13.8 ± 4.61	18.91 ± 4.79	<0.0001
Number of annual AEs *	3.37 ± 1.34	0.97 ± 1.19	<0.0001
Use of OCS (mg/day) *	11.3 ± 9.6	6.5 ± 4.2	<0.0001
FVC (mL) *	3015.21 ± 829.1	3315.17 ± 828.29	0.0018
FEV1 (mL) *	2283.27 ± 721.5	2602.04 ± 701.12	<0.0001
FEV1% *	72.75 ± 17.85	80.95 ± 18.17	0.0134
SNOT-22 *	53.42 ± 22.66	39.39 ± 23.13	0.0003
FENO (ppb)	53.35 ± 33.46	57.51 ± 47.59	0.3410
Eosinophils/μL peripheral blood *	541 ± 283	59.19 ± 48.95	<0.0001
Total IgE (IU/mL)	614.53 ± 1196	1016.26 ± 2477.84	0.8520
sIgE *D. pteronyssinus* (kU_A_/L)	49 ± 49.67	43.93 ± 48.94	0.37
sIgE *D. farinae* (kU_A_/L)	40.17 ± 46.52	41.47 ± 48.07	0.2010
sIgE *Blomia tropicalis* (kU_A_/L)	3.67 ± 15.98	2.88 ± 9.37	0.2691

ACT: Asthma Control Test. AEs: Asthma exacerbations. OCS: Oral corticosteroids. FVC: Forced Ventilatory Capacity. FEV1: forced expiratory volume in the first second. SNOT-22: Sino-Nasal Outcome Test-22. FENO: Fractional exhaled nitric oxide. sIgE: Specific IgE. Mean values and standard deviation are shown. (*) Indicates statistical significance (*p* < 0.05).

**Table 3:** Change from baseline variables in patients with refractory severe asthma with the eosinophilic–allergic phenotype, week-52 after mepolizumab by blood eosinophil count (cells μL) -<600 (*n* = 42) vs. ≥600 (*n* = 19)- categories.

Variables	Baseline	After 52-Weeks of Mepolizumab	*p*
ACT * (<600)	12.38 ± 4.66	18.03 ± 5.27	<0.0001
ACT * (≥600)	14.63 ± 4.19	20.41 ± 3.43	<0.0001
Annual AEs * (<600)	3.33 ± 1.49	1.06 ± 1.38	<0.0001
Annual AEs * (≥600)	3.47 ± 0.96	0.8 ± 0.73	<0.0001
FEV1 * (<600)	2236.19 ± 764.3	2400.64 ± 730.59	0.002
FEV1 * (≥600)	2387.36 ± 622	2933.76 ± 476.09	0.0002
FENO (<600)	47.6 ± 20.81	41.42 ± 28.75	0.4
FENO (≥600)	67.75 ± 53.72	79.21 ± 60.88	0.84

ACT: Asthma Control Test. AEs: Asthma exacerbations. FEV1: Forced expiratory volume in the first second shown in mL. FENO: Fractional exhaled nitric oxide shown in ppb. sIgE: Specific IgE. Mean values and standard deviation are shown. (*) Indicates statistical significance (*p* < 0.05).

**Table 4:** Subgroup analysis of severe asthma patients with refractory severe asthma with the eosinophilic–allergic phenotype associating nasal polyposis (*n* = 22) at baseline and after 52-week treatment with subcutaneous mepolizumab100 mg every 4-week. Nine of 22 included subjects (40.9%) associated a diagnosis of NSAID-exacerbated respiratory disease (NERD).

Variables	Baseline	After 52-Weeks of Mepolizumab	*p*
ACT *	15 ± 4.08	22.33 ± 4.08	<0.0001
Number of annual AEs *	3.04 ± 1.13	0.53 ± 0.7	<0.0001
Use of OCS (mg/day) *	10.21 ± 8.2	4.6 ± 3.9	<0.0001
Number of nasal polyp surgeries *	1.4 ± 0.9	0.22 ± 0.42	<0.0001
FVC (mL) *	3473.54 ± 866.5	3815.66 ± 948.45	0.0082
FEV1 (mL) *	2608.18 ± 746.7	2965.46 ± 708.79	0.0004
FEV1%	77.77 ± 15.53	85.28 ± 17.63	0.073
SNOT-22 *	63.75 ± 20.31	33.5 ± 20.92	<0.0001
FENO (ppb)	58.9 ± 22.61	70.8 ± 44.77	0.711
Eosinophils/μL peripheral blood *	591 ± 322	62 ± 47.14	<0.0001
Total IgE (IU/mL)	295.44 ± 344.05	325.09 ± 366.65	0.124
sIgE *D. pteronyssinus* (kU_A_/L)	1.71 ± 3.77	5.78 ± 11.59	0.224
sIgE *D. farinae* (kU_A_/L)	1.03 ± 2.62	3.8 ± 7.18	0.776
sIgE *Blomia tropicalis* (kU_A_/L)	0	0	0.385

ACT: Table 4: Asthma Control Test. Aes: Asthma exacerbations. OCS: Oral corticosteroids. FVC: Forced Ventilatory Capacity. FEV1: forced expiratory volume in the first second. SNOT-22: Sino-Nasal Outcome Test-22. FENO: Fractional exhaled nitric oxide. sIgE: Specific IgE. Mean values and standard deviation are shown. (*) Indicates statistical significance (*p* < 0.05).

**Table 5:** Adverse drug-related (mepolizumab) events.

Variable	*n* (%)
Number of patients with adverse events related to mepolizumab	3 (4.91)
Musculoskeletal disorders	2 (3.27)
Arthromyalgia	2 (3.27)
Drug administration site disorders	1 (1.63)
Local pain after subcutaneous injection	1 (1.63)
Nervous system disorders	1 (1.63)
Headache	1 (1.63)
Gastrointestinal disorders	1 (1.63)
Dypepsia	1 (1.63)
Patients with adverse events leading to treatment discontinuation	2 (3.27)
Serious adverse events	0 (0%)

## Data Availability

The data that support the findings of this study are available from Servicio Canario de Salud, however, restrictions apply to the availability of these data, which were used under license for the current study, and so are not publicly available. Data are however available from the authors upon reasonable request and with the permission of Servicio Canario de Salud.
